# Evolution of a TRIM5-CypA Splice Isoform in Old World Monkeys

**DOI:** 10.1371/journal.ppat.1000003

**Published:** 2008-02-29

**Authors:** Ruchi M. Newman, Laura Hall, Andrea Kirmaier, Lu-Ann Pozzi, Erez Pery, Michael Farzan, Shawn P. O'Neil, Welkin Johnson

**Affiliations:** 1 Department of Microbiology and Molecular Genetics, New England Primate Research Center, Harvard Medical School, Southborough, Massachusetts, United States of America; 2 Division of Microbiology, New England Primate Research Center, Harvard Medical School, Southborough, Massachusetts, United States of America; 3 Division of Comparative Pathology, New England Primate Research Center, Harvard Medical School, Southborough, Massachusetts, United States of America; 4 Division of Tumor Virology, New England Primate Research Center, Harvard Medical School, Southborough, Massachusetts, United States of America; University of Geneva, Switzerland

## Abstract

The TRIM family proteins share a conserved arrangement of three adjacent domains, an N-terminal RING domain, followed by one or two B-boxes and a coiled-coil, which constitutes the tripartite-motif for which the family is named. However, the C-termini of TRIM proteins vary, and include at least nine evolutionarily distinct, unrelated protein domains. Antiviral restriction factor TRIM5α has a C-terminal B30.2/SPRY domain, which is the major determinant of viral target specificity. Here, we describe the evolution of a cyclophilin-A encoding exon downstream of the *TRIM5* locus of Asian macaques. Alternative splicing gives rise to chimeric transcripts encoding the TRIM motif fused to a C-terminal CypA domain (TRIM5-CypA). We detected TRIM5-CypA chimeric transcripts in primary lymphocytes from two macaque species. These were derived in part from a *CypA* pseudogene in the *TRIM5* locus, which is distinct from the previously described *CypA* insertion in *TRIM5* of owl monkeys. The *CypA* insertion is linked to a mutation in the 3′ splice site upstream of exon 7, which may prevent or reduce expression of the α-isoform. All pig-tailed macaques (*M. nemestrina*) screened were homozygous for the *CypA* insertion. In contrast, the CypA-containing allele was present in 17% (17/101) of rhesus macaques (*M. mulatta*). The block to HIV-1 infection in lymphocytes from animals bearing the *TRIM5-CypA* allele was weaker than that in cells from wild type animals. HIV-1 infectivity remained significantly lower than SIV infectivity, but was not rescued by treatment with cyclosporine A. Thus, unlike owl monkey TRIMCyp, expression of the macaque TRIM5-CypA isoform does not result in increased restriction of HIV-1. Despite its distinct evolutionary origin, *Macaca* TRIM5-CypA has a similar domain arrangement and shares ∼80% amino-acid identity with the TRIMCyp protein of owl monkeys. The independent appearance of TRIM5-CypA chimeras in two primate lineages constitutes a remarkable example of convergent evolution. Based on the presence of the *CypA* insertion in separate macaque lineages, and its absence from sooty mangabeys, we estimate that the *Macaca* TRIM5-CypA variant appeared 5–10 million years ago in a common ancestor of the Asian macaques. Whether the formation of novel genes through alternative splicing has played a wider role in the evolution of the TRIM family remains to be investigated.

## Author Summary

The *TRIM5* gene encodes TRIM5α, a protein that blocks infection of the cell by retroviruses. We previously found that the TRIM5α protein of old world monkeys was highly polymorphic. Here, we describe a substitution in a highly conserved, non-coding element normally required for correct splicing of TRIM5α messenger RNA. While it is difficult to prove positive selection for a non-coding change, the frequency of this mutation in two different species of Asian monkeys (*Macaca sp*) raised the possibility that the mutation was once evolutionarily advantageous. As it turns out, monkeys carrying this substitution also carry a nearby cyclophilin-A (CypA) pseudogene, and these individuals express chimeric mRNA encoding a fusion between the TRIM5 and CypA sequences. Thus, the mutation, which interferes with expression of the normal TRIM5α protein, instead contributes to expression of a novel protein. Remarkably, this is the second example of the appearance of a TRIM5/CypA chimera during primate evolution, the other having occurred in a new world monkey lineage (*Aotus sp*). Cellular CypA binds to the capsid proteins of several lentiviruses, and we believe that TRIM5-CypA proteins were at one time selected for the ability to block infection by retroviral pathogens, possibly related to modern lentiviruses.

## Introduction

The primate TRIM5α protein poses an intrinsic barrier to retroviral replication, blocking infection at an early, post-entry stage in the viral replication cycle [1]. TRIM5 homologues are present in multiple primate lineages [2],[3], including humans and apes, old world monkeys (Asian and African) and new world monkeys (South American), as well as in other mammalian species, including cows [4],[5] and rabbits [6]. A high degree of *TRIM5* sequence divergence between primate species has been reported, as well as evidence for positive selection operating on TRIM5α subdomains responsible for determining target specificity [2],[3]. The *TRIM5* gene of rhesus macaques and sooty mangabeys is highly polymorphic [7], while the human locus may have experienced a reduction in diversity, possibly due to a selective sweep [8].

Owl monkeys (*Aotus sp*) have an unusual *TRIM5* locus, containing a retrotranspositional insertion of a cyclophilin A (*CypA*) pseudogene into the short intron separating the 7th and 8th exons [9],[10]; as a result, owl monkey cells express a TRIM5-CypA fusion protein (TRIMCyp). Because cellular CypA binds to HIV-1 capsid (CA) [11]–[13], the TRIMCyp fusion protein can block HIV-1 infection via an interaction between the CypA domain of TRIMCyp and the incoming viral capsid [14]–[16]. The block to HIV-1 infection of cells expressing owl monkey TRIMCyp can also be overcome by treatment of target cells with the anti-CypA drug cyclosporine A (CsA) [9],[10],[17]. The TRIMCyp variant is present in multiple species within the *Aotus* genus, and is thought to have arisen in a common ancestor of extant owl monkeys between 4.5 and 22 million years ago [18]. Given the well-established antiretroviral activity of TRIM5α ([1] and reviewed in [19]–[23]), the ability of CypA to interact with the CA proteins of several lentiviruses [24], and the capacity of TRIMCyp to block replication of HIV-1, SIVagm and FIV [9],[10],[14], it is possible that fixation of the *CypA* insertion in the *Aotus* lineage was driven by positive selection in the face of infection by an unknown lentiviral pathogen.

The owl monkey *CypA* insertion has not been found in other primate species, including other new world primates related to owl monkeys [9],[18]. Here we report that a distinct, but strikingly similar, TRIM5-CypA gene evolved independently in Asian monkeys of the genus *Macaca*, sometime in the last 5 million to 10 million years. The appearance of two such similar genes during primate evolution stands as a remarkable example of convergent evolution.

## Results

**Figure 1:**
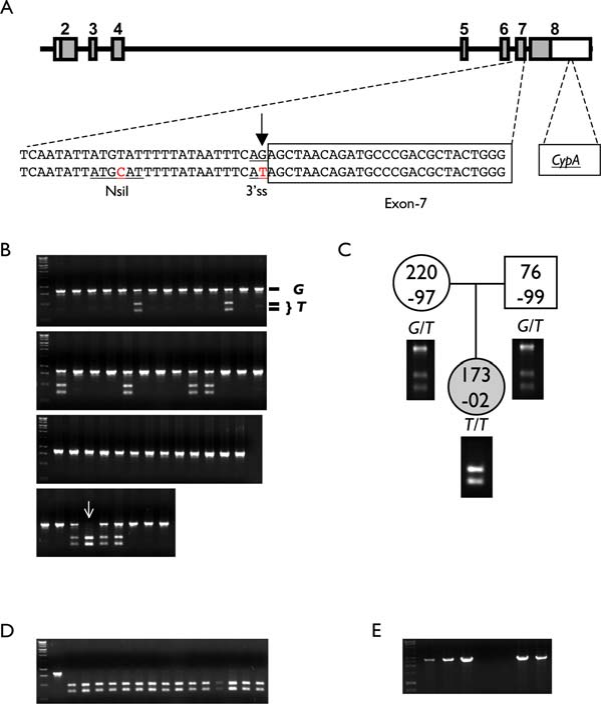
The macaque *TRIM5* locus. A. Schematic depiction of the primate *TRIM5* locus including the seven coding exons (grey shaded regions) and introns, and the nucleotide sequence in the region of the 3′ss G/T SNP at the terminus of intron 6. Sequencing analysis confirmed that an NsiI restriction site was linked to the G/T change, and PCR amplification followed by NsiI digestion was used as an allelic discrimination assay to survey multiple individuals from two species of macaque. B. PCR/NsiI allelic discrimination in rhesus macaques (*M. mulatta*). C. Pedigree depicting genotype of rhesus macaque 173-02 (homozygous T/T) along with its dam (220-97; heterozygous G/T) and sire (76-99; heterozygous G/T). D. PCR+NsiI screening of sixteen Pig-tailed macaques (*M. nemestrina*). E. A second PCR screen of genomic DNA samples for the presence of a *CypA* insertion downstream of TRIM5. A gel revealing the presence of the insertion in 173-02 (T/T), 220-97(G/T), and 76-99 (G/T) (lanes 2–4) is shown. The insertion was not found in two wild type (G/G) individuals (lanes 5 and 6), but was present in two pig-tailed macaques (both T/T) (lanes 7 and 8).

We have previously reported that the TRIM5 coding sequence of old world monkeys is highly polymorphic [7]. In the course of genotyping the *TRIM5* locus in a colony of captive bred rhesus macaques, we identified a single-nucleotide polymorphism in the terminal nucleotide of intron 6 (Figure 1). The SNP is the result of a G-to-T substitution that alters the canonical 3′ splice acceptor site (AG to AU) immediately upstream of exon 7. Initial sequence data revealed the presence of this mutation in 2 of 8 animals, including one homozygote (T/T) and one heterozygote (G/T). The *cis*-acting AG element at the end of introns is a highly conserved feature of 3′ splice sites, and the presence of such a mutation is predicted to interfere with mRNA splicing.

A closely linked substitution in intron-6, 16 nucleotides upstream of the 3′ss, creates a recognition site for the bacterial NsiI restriction endonuclease. The NsiI restriction site polymorphism thus permitted rapid genotyping of a large number of additional animals for the possible presence of the linked 3′ss G/T SNP. Archived genomic DNA samples from 101 animals were chosen at random and genotyped by PCR amplification and NsiI digest; of these, 84 were predicted to be wild type (G/G), 16 heterozygous (G/T) and one was homozygous for the mutation (T/T). The genotypes of eight putative heterozygotes and a single homozygote were confirmed by direct sequencing of PCR products. The observed frequency of the *T* allele was 8.9% (genotypes were 84 G/G, 16 G/T, and 1 T/T). We also genotyped the sire and dam of animal 173-02 (which was homozygous for the minor allele (*T/T*)). Both parents carried the T allele (both were G/T heterozygotes), as would be predicted if the G and T sequences were segregating as alleles of a single locus (Figure 1). It is therefore unlikely that the *T* allele is derived from another *TRIM* gene, a duplication of the *TRIM5* locus, or a repetitive element with chance similarity to intron 6 of the true *TRIM5* locus.

In parallel, genotyping of sixteen pig-tailed macaques (*Macaca nemestrina*) revealed the presence of the identical substitution in this species. Surprisingly, all sixteen animals were homozygous for the 3′ss G-to-T substitution (*T/T*), suggesting that the mutation may be fixed in *M. nemestrina*. Although unlikely, it is also possible that these animals are descended from a small founder population in which the *T* allele was present at high frequency. Recently, another group reported that this same mutation does, in fact, result in aberrant splicing of TRIM5 mRNA transcripts in pig-tailed macaques [25]. In that study, all fourteen animals were also reported to carry the *T* allele. In concordance with their findings [25], we also found that TRIM5 α-isoform transcripts in these animals were the result of aberrant splicing and did not restrict HIV-1 or N-tropic MLV (Figure S2).

**Figure 2:**
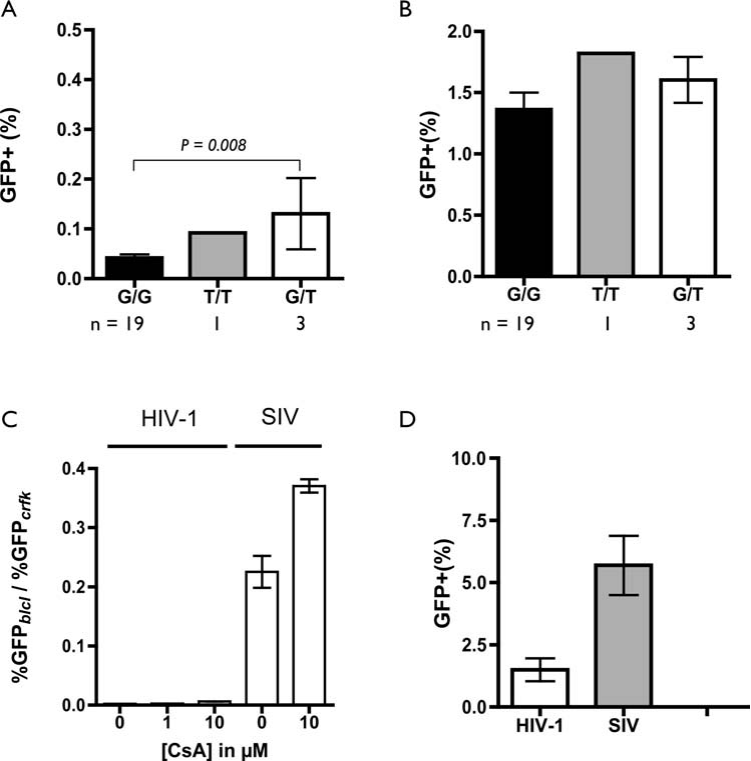
Single-cycle infection assays. Cells were infected at a low M.O.I., to reduce possible effects of saturation. Virus stocks were first titered by serial dilution and infection of CRFK cells (Figure S1), and equivalent infectious units of HIV-1 and SIV were used for parallel infections of macaque PBMC. All experiments were performed in triplicate. A. Infection of activated PBMC from 23 rhesus macaques, including 19 G/G, 1 T/T and 3 G/T individuals, with VSV-pseudotyped HIV-1. B. Same as in A, but using VSV-pseudotyped SIVmac239. C. Single cycle HIV and SIV infection of BLCL derived from a rhesus macaque homozygous for the TRIM5-CypA allele, in the absence and presence of cyclosporine A. D. Single cycle infectivity on immortalized BLCL lines from seven pig-tailed macaques. The BLCL line from each individual animal was tested in triplicate with each of the two viruses. Bars indicate mean infectivity +/−SEM for all seven cell lines.

To begin to test the effects of *TRIM5* polymorphisms on viral infectivity, fresh blood samples were obtained from 22 rhesus macaques that were in the process of undergoing routine veterinary examination at the New England Primate Research Center. In addition, whole blood was obtained from animal 173-02, the previously identified *T/T* homozygote described above. PHA-activated, IL-2 stimulated lymphocytes were prepared from these samples and used as target cells for single-cycle infectivity measurements, using VSV-pseudotyped, HIV-1 and SIV particles carrying a transducible EGFP reporter construct (Figure 2). Uninfected PBMC aliquots from each animal were used to prepare genomic DNA. The genotypes of the donor animals were then determined, and found to include 19 wild-type homozygotes (*G/G*) and three heterozygotes (*G/T*), in addition to the previously typed animal 173-02 (*T/T*). Mean infectivity (% GFP-positive cells) was significantly different between PBMC of wild type homozygotes (*G/G*) and PBMC from animals carrying at least one copy of the T allele (*G/T* and *T/T*) (0.04%+/−0.007% vs 0.12%+/−0.05; p = 0.0068; unpaired, two-tailed t test). The difference remained significant even if the single T/T individual was excluded (0.04%+/−0.007 vs 0.13%+/−0.07; p = 0.0080). Infectivity of VSV-pseudotyped SIV was measured in parallel, and no significant difference in infectivity was found for the different genotypes, consistent with evidence that TRIM5α has little or no restricting activity against SIV (1.36% for G/G vs 1.66% for G/T+T/T; p = 0.3490). However, even in cells from individuals bearing a *T* allele (*G/T* and *T/T*), the mean infectivity of HIV-1 was still substantially lower than that of SIV. This may indicate that sufficient TRIM5α is expressed in these cells, or alternatively, that other post-entry blocks to HIV-1 infection are present.

The presence of a putatively debilitating mutation in a conserved 3′ss element at high frequency in two species is surprising, and suggests that the substitution may have had positive functional consequences for the host. Inspection of the annotated rhesus macaque genome revealed the presence of CypA-related sequences downstream of TRIM5, and in the same transcriptional orientation. To ask whether the G-to-T substitution in the intron-6 3′ss could lead to formation of a hybrid TRIM5-CypA transcript by alternative splicing, we screened several animals of different genotypes by RT-PCR, using a forward primer derived from the beginning of the TRIM5α ORF and a reverse primer corresponding to a conserved region of CypA. A strong band of approximately 1.5 Kb was readily amplified from cellular RNA of rhesus macaques 173-02 (T/T) and 210-02 (*G/T*); we failed to detect this fragment using RNA from homozygous wild type (*G/G*) individuals (n = 4). Thus, expression of these transcripts correlates with the presence of at least one copy of the T allele (G/T or T/T genotypes).

**Figure 3:**
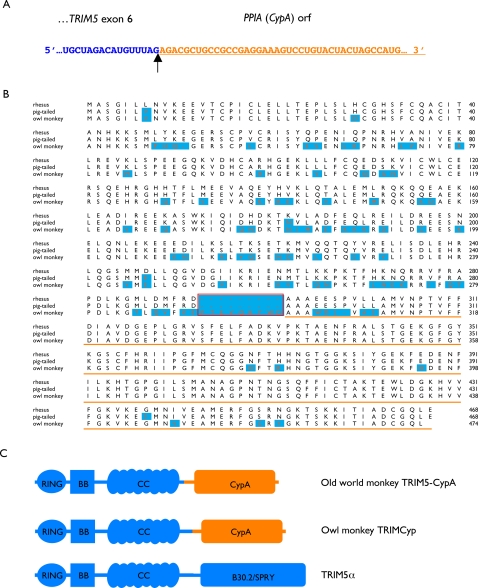
A TRIM5-CypA transcript of macaques is generated by alternative splicing. A. Messenger RNA sequence spanning the junction between the TRIM5 exon-6 and CypA sequences. B. Predicted amino-acid sequence of rhesus TRIM5-CypA aligned with pig-tailed macaque TRIM5-CypA and owl monkey TRIMCyp. Residues that differ from rhesus TRIM5-CypA are highlighted in blue. The region encoded by exon-7 is boxed in red; as the result of alternative splicing, this sequence is present in owl monkey TRIMCyp, but is missing from old world monkey TRIM5-CypA. C. Cartoon depicting predicted protein sequences of the old world monkey TRIM5-CypA protein (top), the owl monkey TRIM-Cyp protein (middle) and wild type primate TRIM5α (bottom).

The RT-PCR product from animal 173-02 was cloned and multiple, insert-containing clones were sequenced. For every clone analyzed, the insert sequence was predicted to encode a TRIM5-CypA fusion protein. Furthermore, in every case, the demarcation between TRIM5 and CypA sequences occurred precisely at a known mRNA splice site, indicating that hybrid transcripts were not artifacts generated by RT-PCR. Two types of transcripts were detected. In some clones (n = 6), the hybrid transcript was formed by splicing from the 3′ terminus of TRIM5 exon 4 to a CypA ORF. The 5′ splice site of exon 4 follows the first nucleotide in a codon, and splicing to the CypA 3′ss from exon 4 results in a frame shift relative to the CypA sequence and a stop codon soon after the splice junction. As a result, the predicted protein product of these transcripts is almost identical to the TRIM5 ε-isoform, except for the addition of 11 C-terminal amino acids derived from the *CypA* insertion. The remaining clones (n = 4) were formed by mRNA splicing between the 3′ terminus of exon 6 and the CypA ORF, and resulted in a single 468 amino-acid open reading frame extending from the TRIM5 AUG initiation-codon, to a UAA stop-codon at the end of the CypA ORF (Figure 3). These results are consistent with a mechanism whereby the G-to-T substitution in the intron-6 3′ss suppresses splicing to exon 7 and promotes alternative splicing to a downstream CypA coding sequence.

Surprisingly, the nucleotide sequence of the CypA ORF in the TRIM5-CypA chimeric transcripts was not identical to any of the CypA sequences proximal to *TRIM5* in the rhesus macaque reference sequence (Mmul_051212), which would be expected if one of these were the source of the *CypA* sequences in the chimeric transcripts. In order to identify the origin of the downstream *CypA* exon, genomic DNA samples from 5 rhesus macaques (one T/T homozygote, 2 G/T heterozygotes and 2 G/G homozygotes) and from two pig-tailed macaques (both homozygous T/T) were used as templates for PCR, using a forward primer derived from exon 6 of the rhesus *TRIM5* gene and a reverse primer corresponding to the 3′ end of cyclophilin A. A single band of approximately 2.5 Kb was amplified from all 3 rhesus macaque samples carrying the 3′ss T substitution, as well as from both pig-tailed macaque samples (Figure 1E). The band was not detected using samples from either of the 2 rhesus G/G homozygotes as templates. Five additional G/T heterozygotes and 29 G/G homozygotes were screened, and the putative CypA insert was only detected in the heterozygous individuals (data not shown). Finally, primers flanking the insertion site were used to screen the same samples for the presence or absence of the insert. In this case, alleles could be discriminated on the basis of size, with the presence of the insert resulting in a band of approximately 2.5 kb and absence of the insert resulting in a band of approximately 2.0 kb. Rhesus macaque 173-02 (T/T) and both pig-tailed macaque samples yielded a single band consistent with the presence of two copies of the CypA+ allele, whereas G/T heterozygotes, including the sire and dam of animal 173-02, yielded two bands; all of the G/G homozygotes yielded a single band of 2.0 kb (Figure S3). Thus, there was an absolute correlation between presence of the G-to-T substitution in the intron-6 3′ss and the presence of the inserted CypA pseudogene.

The amplified fragment was cloned and multiple clones were sequenced on both strands. Analysis of these sequences revealed the presence of an intronless, *CypA*-pseudogene inserted 920 nucleotides downstream of the TRIM5α stop codon in exon 8 of *TRIM5*. The *CypA* insert is not present in the current rhesus macaque whole genome assembly (Mmu1_051212; rheMac2). A continuous 533 bp stretch of the inserted sequence (excluding the PCR primer target sequence) was identical to the *CypA* portion of the hybrid transcripts cloned by RT-PCR, confirming this as the source of the *CypA* sequence present in the cDNA clones. A BLAST query of the nonredundant nucleotide database also identified three unpublished sequence entries described as TRIM5-CypA mRNA from pig-tailed macaques (accession #DQ308404-DQ308406) [26]. The rhesus macaque and pig-tailed macaque TRIM5-CypA amino-acid sequences were 99% identical, while the predicted proteins from both macaque species shared only 81% identity with the TRIMCyp protein of owl monkeys (Figure 3).

We next sought to determine whether the TRIM5-CypA variant was present in other old world primates. Sooty mangabeys (*Cercocebus atys*) are an African species related to the Asian macaques, and the age of the most recent common ancestor of sooty mangabeys and macaques has been estimated at ∼10 million years [27],[28]. To determine whether the TRIM5-CypA allele was present in this species, genomic DNA and cellular RNA samples were extracted from lymphocytes taken from 12 individual sooty mangabeys. RT-PCR failed to detect TRIM5-CypA transcripts in any of the 12 cellular RNA samples, and the inserted CypA pseudogene was not detectable by PCR in any of the 12 corresponding genomic DNA samples. Finally, a PCR product stretching from the end of TRIM5 exon 6 to the beginning of exon 8, including all of intron 6, was amplified from each of the 12 sooty mangabey genomic DNA samples, cloned, and multiple clones per individual were sequenced. None of the clones contained the G-to-T substitution in the 3′ss at the end of *TRIM5* intron-6. Likewise, the T-substitution in the intron-6 3′ss was not found in the human SNP database (dbSNP), and neither the substitution nor the CypA insertion were found in the current releases of the human or chimpanzee reference genome assemblies.

## Discussion

The TRIMs constitute a large protein family, with more than 70 known members among mammalian species [29],[30]. Among these, several are known or suspected to be involved in defending the cell against viral infection [22],[30], including TRIM5 [1], TRIM19, TRIM25 [31] and TRIM28 [32]. All TRIMs share a conserved arrangement of three domains, a RING domain, one or two B-boxes, and a coiled-coil, which constitute the canonical tripartite-motif for which the family is named. However, the additional domains C-terminal to the TRIM motif can vary considerably, and TRIMs encoding at least 9 distinct, unrelated C-terminal protein domains have been described [29],[30]. Here, we described the *de novo* acquisition of an alternative C-terminal domain. In this case, retrotranspositional insertion of a *CypA* pseudogene into the 3′ UTR of *TRIM5* resulted in the formation of a new exon, with alternative splicing from 5′ splice donor sites in *TRIM5* to the inserted *CypA* sequence resulting in the formation of TRIM5-CypA chimeric transcripts. A single point mutation in a highly conserved 3′ss dinucleotide (AG to AT) within the *TRIM5* gene affects proper splicing of α-isoform transcripts ([25] and Figure S2), and may therefore represent an adaptation that facilitates alternative expression of the TRIM5-CypA isoform. Neither the substitution nor the CypA insert were detected in genomic DNA from sooty mangabeys (*Cercocebus atys*), a sister group to the Asian macaques. Multiple lines of evidence place the date of the most recent common ancestor of the two lineages at ∼10 million years ago, whereas the split between rhesus macaques and pig-tailed macaques is thought to have occurred ∼5 million years ago, providing upper and lower estimates for the age of the TRIM5-CypA variant [27],[28]. Alternatively, the TRIM5-CypA variant may be much older, but is either a minor allele in sooty mangabeys, or was lost from that lineage. Further screening of African primate species for the 3ss G-to-T mutation and the CypA insertion may help to date more precisely the origins of TRIM5-CypA.

**Figure 4:**
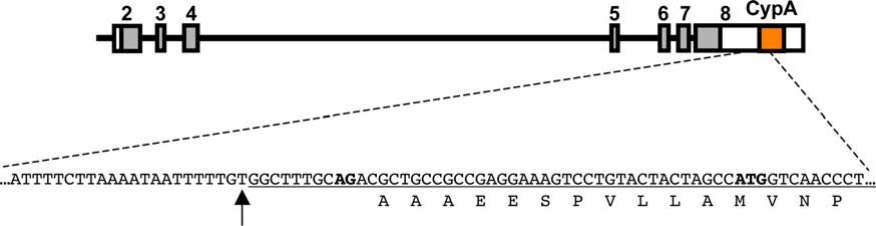
5′ junction of the inserted CypA pseudogene. The first 56 nucleotides of the insertion are underlined. The first nucleotide of the inserted sequence is indicated with an arrow, and occurs just after nucleotide position 671,500 of rhesus macaque chromosome 14 (accession # NW_001100384, based on *M. mulatta* reference assembly Mmu1 01212), in or near the 3′UTR of *TRIM5*. The 3′ss AG dinucleotide and the first methionine codon in the CypA pseudogene are in boldface. Splicing from the end of TRIM5 exon-6 occurs 35 bases upstream of the AUG, but maintains a continuous open reading frame.

Expression of TRIM5-CypA as the result of splicing from TRIM5 to the downstream CypA must also depend on cis-acting splice signals. The alternative 3′ss AG dinucleotide used for generation of chimeric TRIM5-CypA transcripts was present within the inserted *CypA* sequence, and the insertion itself occurred immediately downstream of a pyrimidine-rich tract (Figure 4). Thus, insertion resulted in the juxtaposition of two critical elements (a polypyrimidine tract followed by an AG dinucleotide) that are likely to facilitate formation of the TRIM5-CypA transcripts by alternative splicing [33]. Additionally, a single G-to-T substitution in the 3′splice acceptor upstream of TRIM5 exon-7 (AG to AU in the unprocessed RNA), which we always found linked to the *CypA* insertion, may represent a further adaptation to favor expression of TRIM5-CypA isoforms by preventing or reducing expression of the TRIM5α and TRIM5δ splice-isoforms. However, from the present data, it is not possible to determine whether the G/T substitution in the intron-6 3′ss occurred after insertion of the *CypA* pseudogene, or whether it was already present at the time of insertion.

The predicted proteins encoded by the TRIM5-CypA chimeric transcripts of Asian macaques and South American owl monkeys are remarkably similar (Figure 3). However, unlike owl monkey TRIMCyp, macaque TRIM5-CypA did not block infection by HIV-1. There are multiple nonsynonymous differences between the two proteins, including differences in both the TRIM5 and CypA related domains. In addition, relative to owl monkey TRIMCyp, the macaque variants are missing 9 amino acids corresponding to exon-7 of TRIM5 and perhaps these residues are critical for function of the chimeric protein. It has previously been shown that artificial fusions between CypA and the RBCC domain of rhesus TRIM5α can restrict HIV-1 [15],[34]. Therefore, the functional differences between owl monkey TRIMCyp and macaque TRIM5-CypA (as measured against HIV-1) may instead be due either to differences in the CypA domains, the missing sequences corresponding to exon-7 [35], or both. Some or all of the amino-acid differences, as well as the observed differential restriction of HIV-1, may reflect differences between the natural agents of selection encountered by owl monkey TRIMCyp and macaque TRIM5-CypA during the evolution of each lineage. What those agents were, or if they still exist, is not known and may be impossible to determine. While it would be difficult to prove that the TRIM5-CypA fusions at one time provided (or continue to provide) a selective advantage in nature, the chance appearance of such similar sequences twice during primate evolution, the persistence and ultimate fixation in one genus (*Aotus*) and high frequency in another (*Macaca*)([18],[36] and this study), the known or suspected effects of cellular CypA on lentiviral replication [17], [21], [24], [37]–[39], together with the demonstrable antiviral activity of owl monkey TRIMCyp and various recombinant TRIM5-CypA proteins [9],[10],[14],[15],[34],[40], are compelling arguments that TRIM5-CypA fusions were selected in the face of retroviral pathogens related to modern primate lentiviruses.

New genes are thought to arise in many cases through domain shuffling, as the result of processes such as retrotransposition, segmental duplication, and transcription-induced chimerism (TIC), and several well-characterized examples of each have been reported (reviewed in [41]). Macaque TRIM5-CypA, the consequence of a retrotransposition event coupled to TIC, can now be added to this list. Given that the TRIM protein family is large (>70 known members) with loci spread across multiple chromosomes, and given that individual TRIM genes differ primarily in the nature of their C-terminal domains, it may be that the capture of novel C-terminal domains by alternative splicing has occurred multiple times during the diversification of the TRIM family.

## Materials and Methods

### Nucleic acid isolation

Genomic DNA was isolated from cell lines or lymphocytes (5×10^6^ cells/sample) using the QIAmp DNA Kit (QIAGEN, Inc.) according to the manufacturer's protocol and subsequently used for an allelic discrimination assay or cloned directly into the TOPO TA vector for automated sequence analysis (Retrogen, Inc.). Total RNA was isolated from PBMC or BLCL using the Rneasy Mini Kit; (QIAGEN, Inc.).

### PCR and RT-PCR

For rapid genotyping of samples relative to the G/T polymorphism at the intron-6/exon-7 border, genomic DNA samples isolated from rhesus macaque, pig-tail macaque and sooty mangabey cells served as templates for PCR amplification using primers T5α782F (5′-CATGACCTTGAAGAAGCC-3′) and T5α1087R (5′-GCTTCCCTGATGTGATAC-3′). The resultant 900 bp fragment encompassing all of exon 7 through part of exon 8 of TRIM5 was then digested with the NsiI restriction endonuclease (New England Biolabs). Digestion products were resolved by electrophoresis in a 1.4% agarose gel and visualized with ethidium bromide staining.

To identify the CypA insertion, genomic DNA samples were used as templates for PCR amplification using T5α782F forward primer located in exon 6 of TRIM5 and a reverse primer corresponding to the 3′end of the CypA open reading frame (5′-CGCTCGAGCACAAGTCAAACTTATTCG-3′). Full-length TRIM5-CypA cDNA clones were generated by RT-PCR using primers T5αNotIF (5′-GCGGCCGCATGGCTTCTGGAATC-3′) and CypAR-2 (5′-CGCTCGAGCACAAGTCAAACTTATTCG-3′) and the SuperScript One-Step Kit (Invitrogen). To confirm the presence/absence of the CypA insertion, samples were also screened using the T5α782F forward primer and reverse primer (3′TRIMCyp-2 5′-CAAAATCCTCTCTTCTAGC-3′) corresponding to a target located 39 base pairs downstream of the insertion site. For sequencing, PCR and RT-PCR products were cloned directly into the TOPO TA vector (Invitrogen) and used for sequence analysis.

### Isolation and culture of peripheral blood monocytes

PBMC were isolated from fresh heparinized blood by density centrifugation over LSM medium (ICN Biomedicals). Cells were treated with 2 ug/ml phytohemagglutinin (PHA, Sigma) for 2–3 days, washed and maintained in RPMI/20% FBS containing 10% interleukin-2 (Hemangen Diagnostics, Inc.).

### B-lymphocyte immortalization

Autologous B-lymphoblastoid cell lines (BLCL) were established as previously described [42]. Briefly, B cells were transformed by incubating freshly isolated PBMC with supernatant from the S594 herpesvirus papio producer cell line and propagated in RPMI/20% FBS supplemented with 1 µg/ml cyclosporine A (CsA) and 4 µM AZT. Once established, lines were expanded and aliquoted in the absence of CsA and AZT.

### Single-cycle infectivity assays

Recombinant retroviruses carrying a transducible GFP marker were produced as described [7]. Briefly, HEK293T/17 cells were transfected with appropriate plasmids using the Transfectin Lipid Reagent (BioRad). 72 hours post-transfection, cell-free supernatant was collected and viral titer determined by infection of CRFK cells and subsequent enumeration of GFP+ cells by FACS. Recombinant HIV-1 viruses were produced by cotransfection with pNL43ΔenvFL, pVSV-G (Clontech) and pLenti-GFP. SIVmac recombinant viruses were produced by cotransfection with pHDM.G, pFSΔPRΔINEGFP, and pGPFusion as described in [43]. Plasmids for production of SIVmac recombinant viruses were a gift of David Evans (NEPRC/Harvard Medical School, Southborough). Production of N-tropic MLV (MLV-N) or B-tropic MLV (MLV-B) was carried out by transfection with pCIGN or pCIGB (gift of Jonathan Stoye; MRC, London), along with pVSV-G and pLXIN-EGFP.

For single cycle infectivity assays, 2×10^5^ PBMC or immortalized B-lymphocytes were infected with VSV-pseudotyped virions of HIV-1, SIVmac, MLV-N, or MLV-B. 72 hours post-infection cells were washed, fixed in 3% paraformaldehyde/PBS and expression of EGFP examined by fluorescence-activated cell sorting (FACS). In some experiments, 1 µM or 10 µM cyclosporine A (Sigma) was added to the culture media prior to infection. All FACS experiments were performed on a LSRII flow cytometer (Becton Dickinson) using a 530/30 filter. Data were analyzed using the FlowJo software package (Tree Star, Inc.).

### Accession numbers

Sequences of the two rhesus macaque TRIM5-CypA chimeras were submitted to GenBank (accession numbers EU359036-EU359037) as *Macaca mulatta* clones mmTRIM5-CypA_V1 and mmTRIM5-CypA_V2.

## Supporting Information

Figure S1 **Titer of VSV-pseudotyped HIV-1 and SIV stocks.**
Please see Text S1 for supporting figure legends.
(0.12 MB TIF)

Figure S2 **Point mutation in a 3′ splice site results in formation of aberrant transcripts.**
(0.44 MB TIF)

Figure S3 **PCR screen for CypA insertion.**
(1.37 MB TIF)

Text S1 **Supporting figure legends.**
(0.08 MB DOC)
