# Supplementary figures and images for: Evolution of a TRIM5-CypA Splice Isoform in Old World Monkeys
Source: PLoS Pathog. 2008 Feb 29;4(2):e1000003. doi: 10.1371/journal.ppat.1000003 (PMC2279257; doi:10.1371/journal.ppat.1000003)

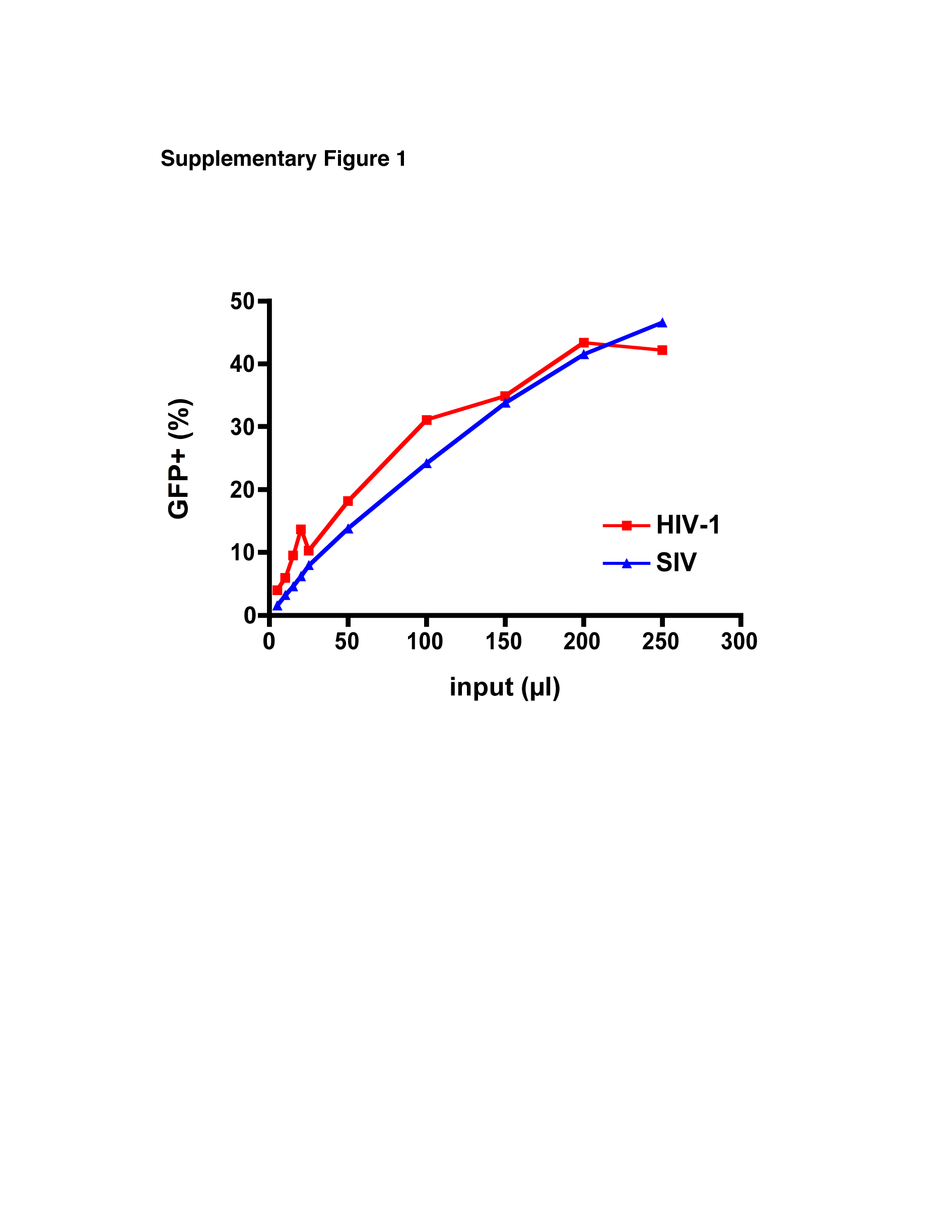

Supplement: Figure S1 — Titer of VSV-pseudotyped HIV-1 and SIV stocks. Please see Text S1 for supporting figure legends. (0.12 MB TIF) [file ppat.1000003.s001.tif]

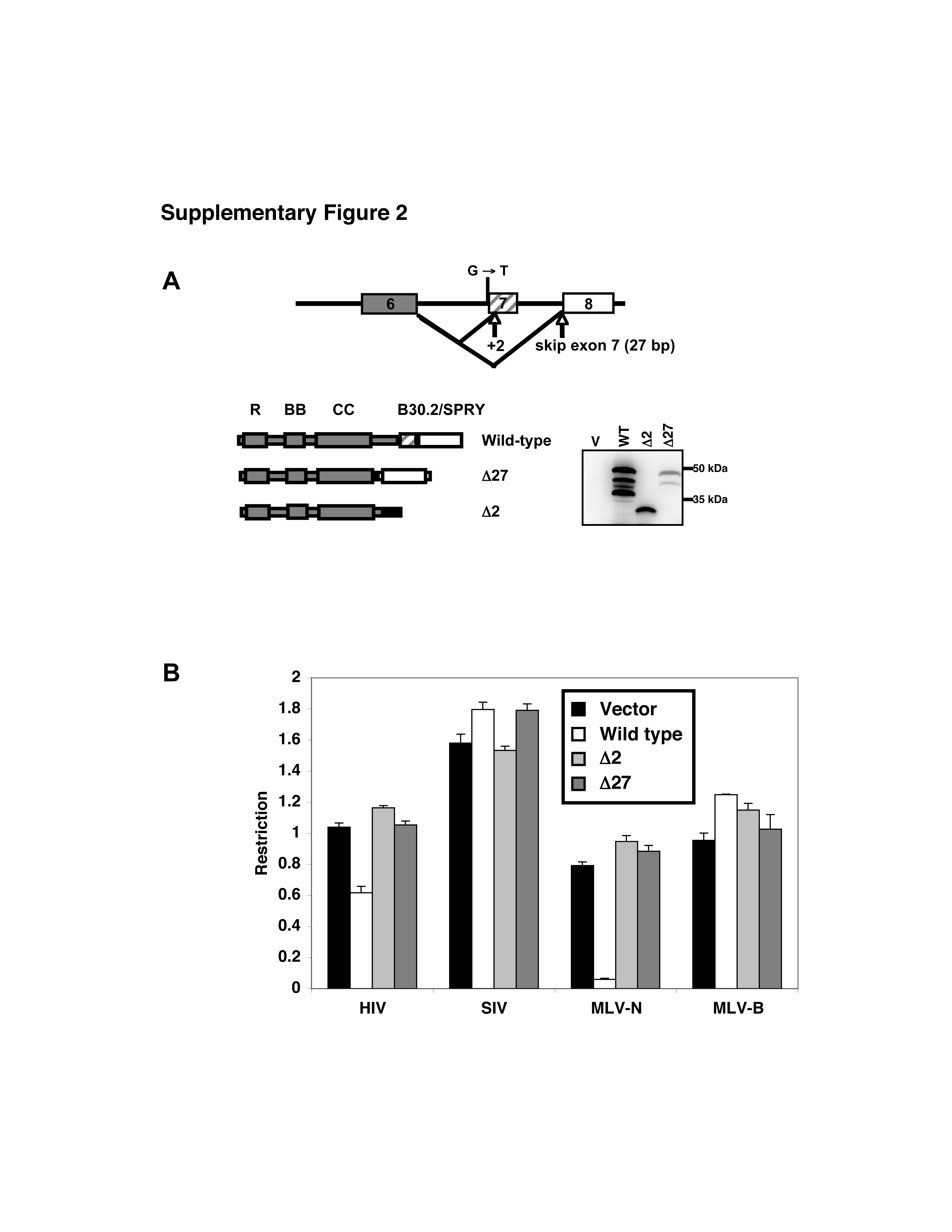

Supplement: Figure S2 — Point mutation in a 3′ splice site results in formation of aberrant transcripts. (0.44 MB TIF) [file ppat.1000003.s002.tif]

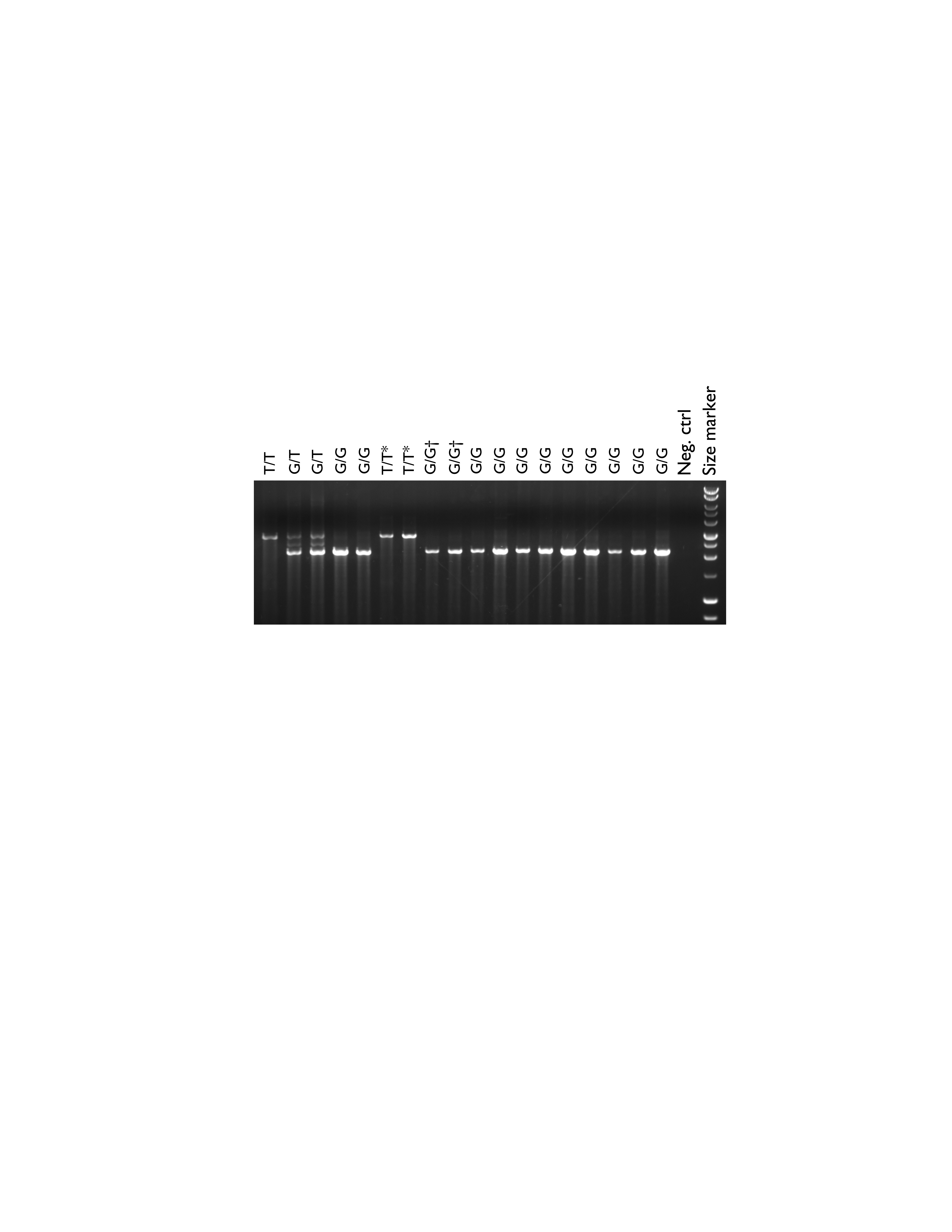

Supplement: Figure S3 — PCR screen for CypA insertion. (1.37 MB TIF) [file ppat.1000003.s003.tif]
