# Supplementary material for: Evolution of a TRIM5-CypA Splice Isoform in Old World Monkeys
Source: PLoS Pathog. 2008 Feb 29;4(2):e1000003. doi: 10.1371/journal.ppat.1000003 (PMC2279257; doi:10.1371/journal.ppat.1000003)
Supplement: Text S1 — Supporting figure legends. (0.08 MB DOC) [file ppat.1000003.s004.doc]

**Supplementary Figure 1.** VSV-pseudotyped HIV-1 and SIV stocks. Virus stocks were produced by transient transfection of HEK293T/17 cells, as described in materials and methods. 72-hours post transfection, supernatant was collected and viral titer was determined by infection of permissive CRFK cells and enumeration of GFP+ cells by flow cytometry. Experiments comparing single-cycle infectivity of HIV-1 and SIV on primary lymphocytes and immortalized B-cell lines were performed using equivalent infectious units, as measured on CRFK cells, as input.

**Supplementary Figure 2.** A common splice acceptor mutant of pig-tailed macaque (*Macaca nemestrina*) TRIM5α gives rise to aberrantly spliced mRNA. Similar results were recently published by Brennan et al [1]. **A**. A high frequency G to T substitution eliminates the 3’ splice site upstream of TRIM5α exon 7 in *M. nemestrina*. Cloning of cDNA from multiple individuals harboring the mutation revealed two forms of aberrant splicing. In some cases, splicing occurred to a cryptic site 2 nucleotides into exon 7, resulting in a two nucleotide deletion at the mRNA level. In this case, the predicted protein is frame-shifted and prematurely truncated (Δ2), and lacks the entire B30.2/SPRY domain. In other cases, splicing skipped the short exon 7 (27 nucleotides), and the predicted protein lacks only 9 amino acids relative to wild-type (Δ27). A western blot comparing recombinant proteins in CRFK cells transduced with TRIM5α-expression constructs is shown. Proteins were detected using a polyclonal anti-TRIM5α antibody (ProSci) and horseradish peroxidase-conjugated anti-IgG (Pierce). Faster migrating species don’t appear in the vector-only control, and are likely to represent degradation products. **B**. Full-length TRIM5α and the splice variants Δ2 and Δ27 were tested for their ability to restrict entry of VSV-pseudotyped HIV-1, SIVmac, MLV-N and MLV-B into permissive CRFK cells, using a previously described two-color flow-cytometry assay for post-entry restriction [2-5].

**Supplementary Figure 3.** Representative results of PCR screening for the presence (~2.5kb bands) or absence (~2.0kb bands) of the CypA insert downstream of rhesus *TRIM5*. Lanes 1-3 include animal 173-02 (T/T) followed by its dam 220-97 (lane 2) and sire 76-99 (lane 3). The remaining samples are from individual rhesus macaques, except those marked with an asterisk (* pig-tailed macaques) or a dagger († sooty mangabeys). The weak, middle band always appears in heterozygote samples and may be the result of heteroduplex formation between the long and short bands.

1. Brennan G, Kozyrev Y, Kodama T, Hu SL (2007) Novel TRIM5 isoforms expressed by Macaca nemestrina. J Virol 81: 12210-12217.

2. Newman RM, Hall L, Connole M, Chen GL, Sato S, et al. (2006) Balancing selection and the evolution of functional polymorphism in Old World monkey TRIM5{alpha}. Proc Natl Acad Sci U S A 103: 19134-19139.

3. Bishop KN, Bock M, Towers G, Stoye JP (2001) Identification of the regions of Fv1 necessary for murine leukemia virus restriction. J Virol 75: 5182-5188.

4. Bock M, Bishop KN, Towers G, Stoye JP (2000) Use of a transient assay for studying the genetic determinants of Fv1 restriction. J Virol 74: 7422-7430.

5. Yap MW, Nisole S, Lynch C, Stoye JP (2004) Trim5alpha protein restricts both HIV-1 and murine leukemia virus. Proc Natl Acad Sci U S A 101: 10786-10791.
